# Unusual Extramedullary Plasmacytoma: A Rare but Possible Cause of Lymphadenopathy in Chronic Lymphocytic Leukemia

**DOI:** 10.1155/2015/657049

**Published:** 2015-07-07

**Authors:** S. P. Chantepie, Q. Cabrera, J. B. Mear, V. Salaun, E. Lechapt-Zalcman, M. Macro

**Affiliations:** ^1^Department of Hematology, Caen University Hospital, 14000 Caen, France; ^2^Hematology Laboratory, Caen University Hospital, 14000 Caen, France; ^3^Department of Pathology, Caen University Hospital, 14000 Caen, France

## Abstract

Cervical bilateral lymphadenopathy is a frequent event during chronic lymphocytic leukemia (CLL) natural history. However, lymph node biopsy is generally not required as long as transformation into an aggressive lymphoma (Richter syndrome) is not suspected. We present here a rare case of CLL patient who developed progressive bilateral cervical lymph node and bilateral tonsillar hypertrophy. CLL front-line therapy was ineffective leading to adenectomy and diagnosis of concomitant extramedullary plasmacytoma. Radiotherapy did not result in the disappearance of lymphadenopathy. Adenectomy should be performed in CLL cases to avoid misdiagnosis.

## 1. Introduction

Cervical bilateral lymphadenopathy is a frequent event during chronic lymphocytic leukemia (CLL) natural history. CLL diagnosis criteria include immunophenotype of the neoplastic lymphocytes to detect the coexpression of CD5 and B-cell surface antigens CD19, CD20, and CD23 on lymphocyte. However, lymph node biopsy is generally not required as long as transformation into an aggressive lymphoma (Richter syndrome) is not suspected. We present here a rare case of CLL patient who developed progressive bilateral cervical lymph node and bilateral tonsillar hypertrophy leading to diagnosis of concomitant extramedullary plasmacytoma. The clonal origin of the two diseases is also discussed.

## 2. Case Presentation

**Figure 1:**
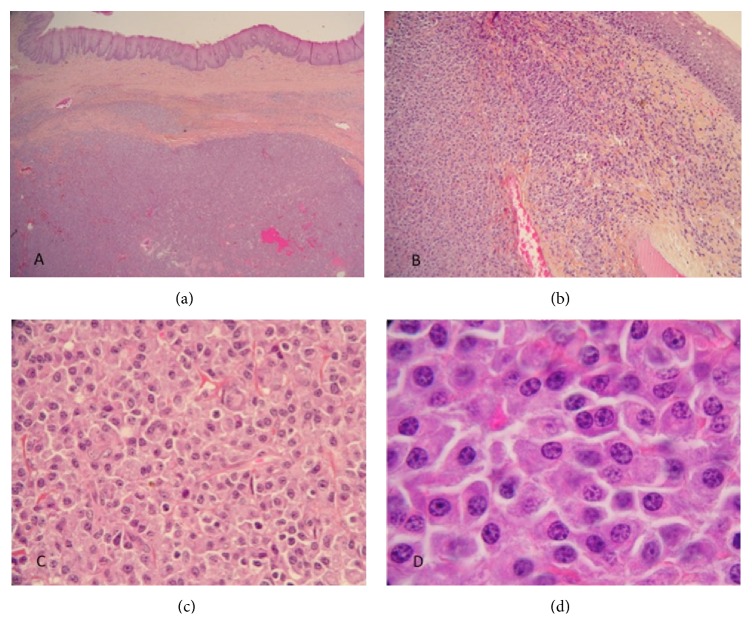
Tonsil biopsy section showing a diffuse and dense infiltrate of a plasma cell proliferation in the subepithelial tissue (hematoxylin-eosin-safran, original magnification ×2 (a) and ×10 (b)). The proliferation consists of mononuclear and multinucleated well-differentiated neoplastic plasma cells (×40 (c) and ×100 (d)).

**Figure 2:**
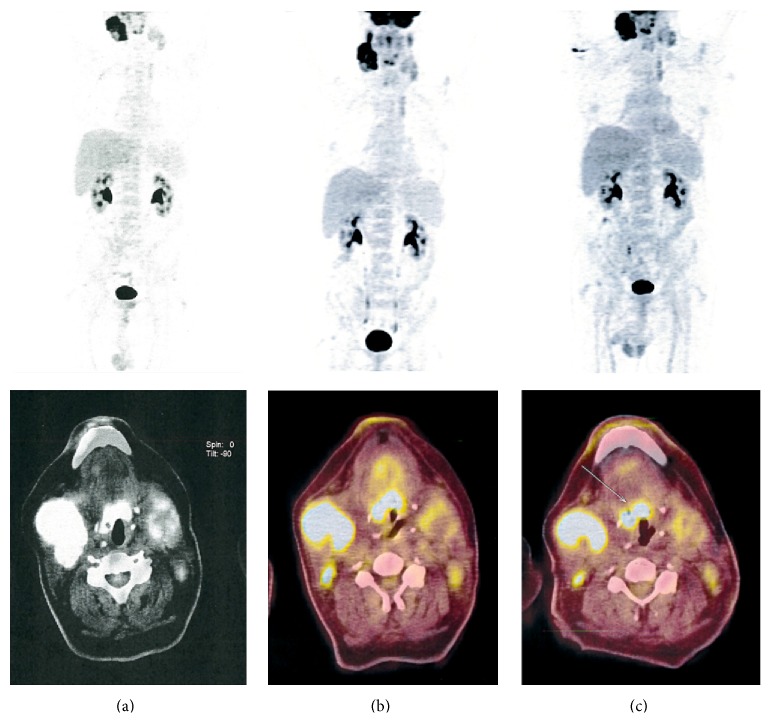
(a) PET-CT before radiotherapy showing bilateral abnormal uptake in cervical bilateral adenopathy and in right tonsil; (b) and (c) PET-CT 2 and 4 months after radiotherapy showing the persistence of FDG uptakes in the previous involved site.

A 63-year-old patient with a history of CLL since 2003 was referred to the Caen University Hospital. Binet's stage was A, and Matutes score was 5. Immunophenotype revealed CD5/CD19/kappa positive CLL cells. He complained of a gradual increase in bilateral cervical lymphadenopathy which measured 10 cm right and 5 cm left with bilateral tonsillar hypertrophy. He did not mention weight loss or night sweat. He also had lymphocytosis (8.5 G/L) and an IgG kappa monoclonal protein (24 g/L). Fludarabine, cyclophosphamide, and rituximab therapy was started without any response on lymphadenopathy after 6 cycles. Four cycles of R-CHOP were also ineffective. Right cervical adenectomy and right tonsillectomy revealed extramedullary plasmacytoma with the same kappa light chain on histological examination (Figure 1) and in flow cytometry analysis compared to the initial circulating CLL clone. Flow cytometry plasma cells were CD38/CD138/CD56/kappa strong and negative for CD19/CD20/CD33/CD117/CD45. No CLL cells were found in nodes and tonsils. The bone marrow biopsy and bone marrow aspiration failed to detect any CLL residual disease or clonal plasma cells. PET-CT scan showed FDG uptake in the right and left cervical area, the base of the tongue, and the right tonsil (Figure 2(a)). Surprisingly, radiotherapy 40 Gy was ineffective (Figures 2(b) and 2(c)). Lymph nodes sizes eventually decreased with bortezomib and dexamethasone therapy leading to a very good partial response on monoclonal component and cervical plasmacytoma disappearance. The patient is still alive 3 years after treatment without any lymphadenopathy but with a low residual monoclonal component (2 g/L).

## 3. Discussion

To date, only 3 other cases of plasmacytoma in CLL patient have been described [1–3], but only one had the same light chain in plasmacytoma and CLL clone [1]. Other plasma cells disorders have been described in the context of CLL, as multiple myeloma or amyloidosis with or without the same light chain in both clones [4, 5]. Several reports suggest that plasma cell clone may derive from CLL cells with the same immunoglobulin production [6]. Immunoglobulin gene rearrangement investigations support the theory of a single clone for two concomitant malignant diseases [7]. However, absence of clonal chromosomal or molecular relationship between concomitant B-CLL and multiple myeloma has been described [8, 9] and recent analysis with single nucleotide polymorphism (SNP) mapping array and FISH analyses on bone marrow (BM) smears supported the hypotheses of biclonal origin [10].

Whatever the clonal origin of the two diseases is, the “take home” message is to perform biopsy in patients with CLL when tumoral lymph nodes do not disappear with conventional therapy. Lymphadenopathy in CLL patient may also be a plasmacytoma.

## 4. Conclusion

Lymphadenopathy in a CLL patient may lead to adenectomy to be sure of CLL diagnosis to avoid misdiagnosis and wrong therapeutic strategy.
